# 10-year spatial and temporal trends of PM_2.5_ concentrations in the southeastern US estimated using high-resolution satellite data

**DOI:** 10.5194/acp-14-6301-2014

**Published:** 2014-06-25

**Authors:** X. Hu, L. A. Waller, A. Lyapustin, Y. Wang, Y. Liu

**Affiliations:** 1 Department of Environmental Health, Rollins School of Public Health, Emory University, Atlanta, GA 30322, USA; 2 Department of Biostatistics & Bioinformatics, Rollins School of Public Health, Emory University, Atlanta, GA 30322, USA; 3 NASA Goddard Space Flight Center, Greenbelt, MD, USA; 4 University of Maryland Baltimore County, Baltimore, MD, USA

## Abstract

Long-term PM_2.5_ exposure has been associated with various adverse health outcomes. However, most ground monitors are located in urban areas, leading to a potentially biased representation of true regional PM_2.5_ levels. To facilitate epidemiological studies, accurate estimates of the spatiotemporally continuous distribution of PM_2.5_ concentrations are important. Satellite-retrieved aerosol optical depth (AOD) has been increasingly used for PM_2.5_ concentration estimation due to its comprehensive spatial coverage. Nevertheless, previous studies indicated that an inherent disadvantage of many AOD products is their coarse spatial resolution. For instance, the available spatial resolutions of the Moderate Resolution Imaging Spectroradiometer (MODIS) and the Multiangle Imaging SpectroRadiometer (MISR) AOD products are 10 and 17.6 km, respectively. In this paper, a new AOD product with 1 km spatial resolution retrieved by the multi-angle implementation of atmospheric correction (MAIAC) algorithm based on MODIS measurements was used. A two-stage model was developed to account for both spatial and temporal variability in the PM_2.5_–AOD relationship by incorporating the MAIAC AOD, meteorological fields, and land use variables as predictors. Our study area is in the southeastern US centered at the Atlanta metro area, and data from 2001 to 2010 were collected from various sources. The model was fitted annually, and we obtained model fitting *R*^2^ ranging from 0.71 to 0.85, mean prediction error (MPE) from 1.73 to 2.50 μg m^−3^, and root mean squared prediction error (RMSPE) from 2.75 to 4.10 μg m^−3^. In addition, we found cross-validation *R*^2^ ranging from 0.62 to 0.78, MPE from 2.00 to 3.01 μgm^−3^, and RMSPE from 3.12 to 5.00 μgm^−3^, indicating a good agreement between the estimated and observed values. Spatial trends showed that high PM_2.5_ levels occurred in urban areas and along major highways, while low concentrations appeared in rural or mountainous areas. Our time-series analysis showed that, for the 10-year study period, the PM_2.5_ levels in the southeastern US have decreased by ∼20 %. The annual decrease has been relatively steady from 2001 to 2007 and from 2008 to 2010 while a significant drop occurred between 2007 and 2008. An observed increase in PM_2.5_ levels in year 2005 is attributed to elevated sulfate concentrations in the study area in warm months of 2005.

## 1 Introduction

Long-term exposure to PM_2.5_ (particle size less than 2.5 μm in the aerodynamic diameter) is associated with various adverse health outcomes including respiratory and cardiovascular diseases (Crouse et al., 2012; Peng et al., 2009). Due to the spatiotemporally continuous nature of the distribution of fine particles, obtaining long-term and spatially resolved distribution of PM_2.5_ concentrations is important to reduce exposure misclassification and facilitate epidemiological studies in the region. In addition, time-series analyses of air pollution and human health have become a common study design to compare day-to-day fluctuations of air pollution and corresponding fluctuations in health outcomes (Ito et al., 2007) and require long-term PM_2.5_ concentration estimates. Previous research examined temporal trends in PM_2.5_ levels. For instance, Weber et al. (2003) investigated the temporal variations in PM_2.5_ concentrations at the US Environmental Protection Agency (EPA) Atlanta Supersite Experiment in August, 1999. So et al. (2007) examined long-term variation in PM_2.5_ levels during two 12-month periods in Hong Kong. The EPA (2011) evaluated temporal trends of annual and 24 h mean PM_2.5_ concentrations at the national level from 2001 to 2010 and reported that annual and 24 h mean PM_2.5_ concentrations dropped 24 and 28 %, respectively, during these 10 years.

Accurate depiction of spatial trends of PM_2.5_ levels is also important for air pollution health effects research. Stationary ambient monitors leave large areas uncovered, which makes the assessment of PM_2.5_ spatial variability difficult. Using measurements from central monitors to estimate population exposure inevitably introduces measurement errors that likely have substantial implications for interpreting epidemiological studies, especially time-series analyses (Zeger et al., 2000). On the other hand, aerosol observations from satellite remote sensing could substantially improve estimates of population exposure to PM_2.5_ (van Donkelaar et al., 2010). As a result, satellite-retrieved aerosol optical depth (AOD), which measures light extinction by aerosols in the atmospheric column, has been widely used to predict ground-level PM_2.5_ concentrations, given its relatively low cost and large spatiotemporal coverage. A number of AOD products from sensors such as the Moderate Resolution Imaging Spectroradiometer (MODIS), the Multiangle Imaging Spectro-Radiometer (MISR), and the Geostationary Operational Environmental Satellite Aerosol/Smoke Product (GASP) have been applied to PM_2.5_ concentration prediction in previous studies (Liu et al., 2007, 2009; Paciorek et al., 2008; Hu et al., 2013). A limitation of those AOD products is the relatively coarse spatial resolutions. For instance, the spatial resolutions of AOD derived from MODIS and MISR are 10 and 17.6 km, respectively. Although GASP has a spatial resolution of 4 km, the AOD retrievals are less precise than those from the polar-orbiting instruments due to limited information content (one spectral band) and relatively low signal-to-noise ratio of the GOES sensor (Prados et al., 2007). Meanwhile, epidemiological studies typically have access to health data geo-coded to small geographical units (e.g., zip code and census block groups), many of which are substantially smaller than the spatial resolutions of MODIS and MISR. In addition, satellite-estimated PM_2.5_ concentrations at coarse resolutions omit detailed spatial variability of PM_2.5_ exposure and therefore have limited value in the investigation of spatial trends of PM_2.5_ levels at urban scale (Hu et al., 2014). Hence, it is essential to use high-resolution AOD retrievals to generate high spatial resolution PM_2.5_ concentration estimates. Recently, a new AOD product retrieved by the multi-angle implementation of atmospheric correction (MAIAC) algorithm based on MODIS measurements has been reported (Lyapustin et al., 2011b). MAIAC AOD has a spatial resolution of 1 km and thus has the ability to estimate PM_2.5_ concentrations at that resolution. Moreover, MAIAC AOD has been demonstrated to be correlated with monitored PM_2.5_ levels in the New England region (Chudnovsky et al., 2012). Hu et al. (2014) compared the performance of MAIAC with MODIS in PM_2.5_ concentration prediction in the southeastern US in a case study and found that MAIAC predictions can reveal many more spatial details than MODIS. In a single 12 × 12 km^2^ Community Multiscale Air Quality (CMAQ) grid cell, MODIS can only make one prediction, while MAIAC can make ∼144 predictions. As an example of the benefit gained with increased resolution, MAIAC predictions can distinctly show high concentrations along major highways, while MODIS predictions cannot.

Various statistical methods have been developed to establish the quantitative relationship between PM_2.5_ and satellite-derived AOD, including linear regression (Schafer et al., 2008; Wallace et al., 2007; Gupta and Christopher, 2009). However, many of the methods do not consider day-to-day variability in the association between PM_2.5_ and AOD. Lee et al. (2011) and Kloog et al. (2011) argued that the PM_2.5_–AOD relationship varies day to day, and this temporal variability needs to be accounted for in order to improve the performance of the AOD-based prediction models. As a result, both studies developed a linear mixed effects (LME) model to incorporate daily calibration of the PM_2.5_–AOD relationship and obtained predictions with high accuracy. To move one step further, Hu et al. (2014) introduced a geographically weighted regression (GWR) model as the second stage to account for possible spatial variability in the PM_2.5_–AOD relationship. This model used the MAIAC AOD as the primary predictor and meteorological fields and land use variables as secondary predictors. Hu et al. (2014) further pointed out that AOD is essential in the two-stage model framework in terms of prediction accuracy. The model can predict PM_2.5_ concentrations with high accuracy and thus was adopted in this study.

The objectives of this paper were, first, to estimate spatiotemporally resolved PM_2.5_ concentrations in the study domain during the period between 2001 and 2010 in the southeastern US using the two-stage model developed by Hu et al. (2014). Second, maps of annual mean PM_2.5_ concentrations as well as the changes between 2001 and 2010 were generated from the daily estimates to visually illustrate the spatial trends of annual PM_2.5_ levels between 2001 and 2010. Third, time-series analyses were conducted for the study domain and the Atlanta metro area specifically using the seasonal and annual mean PM_2.5_ estimates to examine the 10-year temporal trends of PM_2.5_ levels, and the underlying causes were discussed.

## 2 Materials and methods

### 2.1 Study area

**Figure 1:**
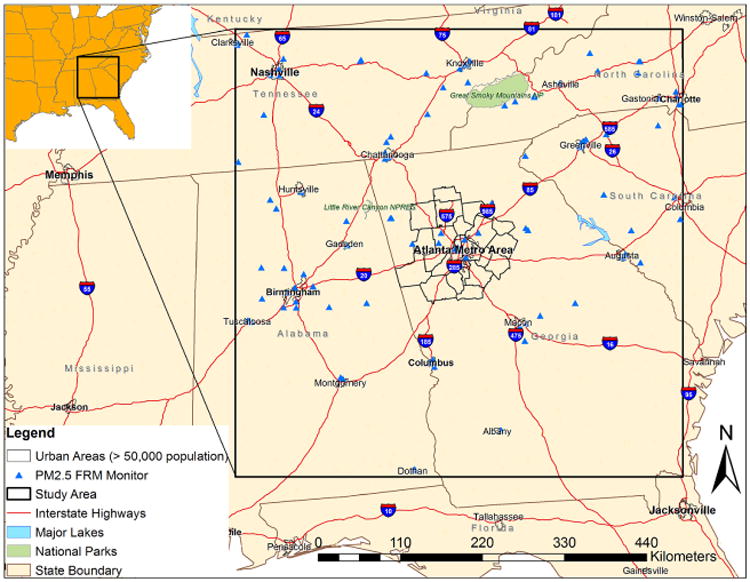
Study area.

The study area is approximately 600 × 600 km^2^ in the southeastern US, covering most of Georgia, Alabama, and Tennessee, and parts of North and South Carolina (Fig. 1). The domain includes several large urban centers, numerous medium-to-small cities, as well as suburban and rural areas.

### 2.2 PM_2.5_ measurements

The 24 h average PM2.5 concentrations from 2001 to 2010 collected from the US EPA federal reference monitors (FRMs) were downloaded from the EPA's Air Quality System Technology Transfer Network (http://www.epa.gov/ttn/airs/airsaqs/). PM2.5 concentrations less than 2μgm^−3^ (**∼** 0.2–3 % of total data records) were discarded as they are below the established limit of detection (EPA, 2008a).

### 2.3 Remote sensing data

MAIAC retrieves aerosol parameters over land at 1 km resolution, which was accomplished by using the time series of MODIS measurements and simultaneous processing of a group of pixels in fixed 25 × 25 km^2^ blocks (Lyapustin et al., 2011a, b, 2012). MAIAC uses a sliding window to collect up to 16 days of MODIS radiance observations over the same area and processes them to obtain surface parameters used for aerosol retrievals. To facilitate the time-series analysis, MODIS data are initially gridded to a 1 km resolution in a selected projection. For this work, we used MODIS level 1B (calibrated and geometrically corrected) data from Collection 6 re-processing, which removed major effects of temporal calibration degradation of Terra and Aqua, a necessary prerequisite for the trend analysis.

Validation based on the Aerosol Robotic Network (AERONET) data showed that MAIAC and the operational Collection 5 MODIS Dark Target AOD have a similar accuracy over dark and vegetated surfaces, but also showed that MAIAC generally improves accuracy over brighter surfaces, including most urban areas (Lyapustin et al., 2011b). MAIAC AOD data from 2001 to 2010 were obtained from the National Aeronautics and Space Administration (NASA) Goddard Space Flight Center. Due to the lack of sufficient data records from AERONET, a comparison between MAIAC AOD and AERONET measurements in our study domain was not possible.

Zhang et al. (2012) found that Terra and Aqua may provide a good estimate of the daily average of AOD. Thus, the average of the Aqua and Terra measurements can be used to predict daily PM2.5 concentrations. In this study, Aqua (overpasses at **∼** 1:30 p.m. local time) and Terra (overpasses at **∼** 10:30 a.m. local time) MAIAC AOD values were first combined to improve spatial coverage. In our study domain, the increase in spatial coverage ranged from 30.2 to 72.4 % for Aqua and from 17.2 to 26.3 % for Terra from 2001 to 2010. In a common MAIAC pixel, there might be only one MAIAC product from either Aqua or Terra, or both may be present. In the second case, when we combine them, the averaged value, as pointed out by Lee et al. (2012), is likely to better reflect daily aerosol loading, yet in the first case, AOD as an indicator of PM_2.5_ abundance is biased towards the atmospheric condition either in the morning or early afternoon. To estimate the missing AOD value, Lee et al. (2011) defined a simple ratio between averaged Terra and Aqua AOD. In this study, we fitted a linear regression to define the relationship between daily mean Terra-MAIAC and Aqua-MAIAC AOD values. We used this regression to predict the missing AOD value (i.e., predict Terra-MAIAC AOD with the available Aqua-MAIAC AOD, and vice versa), and then averaged the observed and the predicted AOD values together. Finally, we set an upper bound of 2.0 for the combined AOD to reduce potential cloud contamination (∼0.05–0.1 % of total data records were excluded).

### 2.4 Meteorological fields

The meteorological fields provided by the North American Land Data Assimilation System (NLDAS) Phase 2 were downloaded from the NLDAS website (http://ldas.gsfc.nasa.gov/nldas/). The spatial resolution of NLDAS meteorological data is 1/8th of a degree (∼ 13 km). Another meteorological data set used in this study is the North American Regional Reanalysis (NARR). NARR is a long-term, consistent, high-resolution climate data set for North America (Mesinger et al., 2006), with a spatial resolution of ∼ 32 km. NLDAS provides most of the meteorological fields used in this analysis, including relative humidity, *U* wind, and *V* wind, while NARR provides another critical parameter: boundary layer height. To generate daytime meteorological fields corresponding to the MODIS overpass times, 3-hourly NARR measurements and hourly NLDAS measurements from 10 a.m. to 4 p.m. standard local time were averaged.

### 2.5 Land use variables

Elevation data were downloaded from the national elevation data set (NED) (http://ned.usgs.gov). NED is the seamless elevation data set covering the conterminous United States and is distributed by the US Geological Survey (USGS). The elevation data are downloaded at a spatial resolution of 1 arcsec (∼30m). The road data were obtained from ESRI StreetMap USA (Environmental Systems Research Institute, Inc., Red-land, CA). The road data at level A1 (limited access highway) were extracted. Summed length of road segments was calculated for each 1 × 1 km^2^ MAIAC grid cell, and grid cells with no roads were assigned zero. The 2001 and 2006 Landsat-derived land cover maps covering the study area with a spatial resolution of 30 m were downloaded from the National Land Cover Database (NLCD) (http://www.mrlc.gov). Forest cover maps were generated by assigning one to forest pixels and zero to others. Primary PM_2.5_ emissions (tons per year) were obtained from the 2002, 2005, and 2008 EPA National Emissions Inventory (NEI) facility emissions reports. Grid cells with multiple emission sources were assigned the summed value, and those with no emissions were assigned zero.

### 2.6 Data integration

All the data were first re-projected to the USA Contiguous Albers Equal Area Conic USGS coordinate system. For model fitting, a 1 × 1 km^2^ square buffer was generated for each PM_2.5_ monitoring site. Meteorological fields and AOD values were assigned to each PM_2.5_ monitoring site using the nearest neighbor approach. Forest cover and elevation were averaged, while road length and point emissions were summed over the 1 × 1 km^2^ square buffer by calculating the total length of road segments and total point emissions within the buffer. For PM_2.5_ prediction, the same procedure was performed for each 1×1km^2^ MAIAC grid cell.

### 2.7 Model structure

We adopted the two-stage spatiotemporal model developed by Hu et al. (2014). For the model to be valid, we assumed that particles within the boundary layer were well mixed, and the vertical distribution of particles above boundary layer was relatively smooth. The first stage is a LME model with day-specific random intercepts and slopes for AOD and meteorological fields to account for the temporally varying relationship between observed PM2.5 and AOD (Eq. 1). The model structure can be expressed as

(1) $${\text{PM}}_{2.5,st}=(b_{0}+b_{0,t})+(b_{1}+b_{1,t}){\text{AOD}}_{st}+(b_{2}+b_{2,t}){\text{Meteorological Fields}}_{st}+b_{3}{\text{Elevation}}_{s}+b_{4}{\text{MajorRoads}}_{s}+b_{5}{\text{ForestCover}}_{s}+b_{6}{\text{PointEmissions}}_{s}+{ɛ}_{st}(b_{0,t}b_{1,t}b_{2,t})\sim N[(0,0,0),\Psi ],$$

where *b_i_* and *b_i_*_,t_ (day-specific) are the fixed and random intercept and slopes, respectively. Fixed intercepts and slopes are the same for all days and generated via conventional linear regression, while random intercepts and slopes vary independently for each individual day and are estimated via likelihood methods from the full set of observations. In this study, we generated fixed slopes for each predictor variable, but random slopes were only generated for AOD and meteorological fields, since they represent time-varying variables. The fixed slopes (e.g., *b*_1_, *b*_2_,…, *b*_6_) denote the overall relationship for all days, and the random slopes (e.g., *b*_1,_*_t_b*_2,_*_t_*) indicate the daily relationship among PM_2.5_, AOD, and meteorological fields. PM_2.5,s_*_t_* is the measured ground level PM_2.5_ concentration (μgm^−3^) at site *s* on day *t*; AOD_st_ is the MAIAC AOD value (unitless) at site *s* on day *t*; Meteorological Fields_st_ is the meteorological parameters at site *s* on day *t* and may include Relative Humidity_s*t*_, Boundary Layer Height_s*t*_, Wind Speed_s*t*_, *U* Wind_s*t*_, and *V* Wind_s*t*_; Relative Humidity_s*t*_ is the relative humidity (%) at site *s* on day *t*; Boundary Layer Heightst is the boundary layer height (m) at site *s* on day *t*; Wind Speed_s*t*_ is the 2 m wind speed (m s^−1^) at site *s* on day *t; U* Wind_s*t*_ is the east-west component of wind (ms^−1^) at site *s* on day *t; V* Wind_s*t*_ is the north–south component of wind (m s^−1^) at site *s* on day *t*; Elevation_s_ is elevation values (m) at site *s*; Major Roads_s_ is road length values (m) at site *s*; Forest Cover_s_ is forest cover values at site *s*; Point Emissions_s_ is point emissions (tons per year) at site *s*; and Ѱ is an unstructured variance–covariance matrix for the random effects. The fixed effects affect the population mean and represent the average effects on PM_2.5_ concentration estimates for the entire period, while the random effects contribute to the covariance structure and account for the daily variability in associations between dependent and independent variables. Although the PM_2.5_–AOD relationship might vary by season, our first-stage LME model was able to incorporate daily variability in the relationship by generating day-specific random slopes for AOD and meteorological fields and thus should be able to capture the seasonal variability. In addition, by comparing the performances of models fitted for each season, each year, and all 10 years, we found that the models fitted for each year generally yielded higher prediction accuracy. Hence, in this study, we fitted the model for each year individually allowing the predictors used in each model to vary from year to year. Each final annual model was selected to achieve the highest prediction accuracy, and only statistically significant variables were retained. The detailed model structures can be found in the Supplement.

The second stage is a geographically weighted regression (GWR) model that can generate a continuous surface of estimates for each parameter at each location instead of a universal value for all observations. We fitted a monthly GWR model to calibrate the spatial variability within the PM_2.5_–AOD relationship, and the model can be expressed as

(2) $${\text{PM}}_{2,5}\_{\text{resi}}_{st}=\beta_{0,s}+\beta_{1,s}{\text{AOD}}_{st}+{ɛ}_{st},$$

where PM_2.5__resi_s*t*_ denotes the residuals from the stage one model at site *s* in month *t*, AOD_s*t*_ is the MAIAC AOD value (unitless) at site *s* in month *t*, and *β*_0,s_ and *β*_1,s_ denote the location-specific intercept and slope, respectively.

To assess the goodness of fit of the model, various statistical indicators such as the coefficient of determination (*R*^2^), mean prediction error (MPE), and square root of the mean squared prediction errors (RMSPE) were calculated between the fitted PM_2.5_ concentrations from the model and the observations. In addition, a 10-fold cross-validation (CV) technique was adopted to assess potential model over-fitting. A model that has been over-fit could perform better on the data used to fit the model than unobserved data and thus generally has poor predictive performance. The entire model-fitting data set was randomly split into 10 subsets with approximately 10 % of the total data records in each subset. In each round of cross-validation, we selected one subset (10 % of the data) as the testing samples and used the remaining nine subsets (90 % of the data) to fit the model. Predictions of the held-out subset (10 % of the data) were made from the fitted model. The process was repeated 10 times until every subset was tested. Statistical indicators such as *R*^2^, MPE, and RMSPE were calculated between the CV predicted concentrations and the observations. The model over-fitting assessment was conducted by comparing the CV and model-fitting statistics. Cross-validation also can provide a means to quantitatively assess prediction accuracy for areas where there are no ground observations. A relative accuracy value was also calculated for each year to make validation results comparable among different years.

The daily PM_2.5_ concentrations were estimated using the final annual models for 2001 through 2010. The maps of annual mean PM_2.5_ concentrations as well as the percent changes between 2001 and 2010 for the study domain and the Atlanta metro area were generated using the daily estimates to visually examine spatial trends of PM_2.5_ levels between 2001 and 2010. The percent changes were calculated as follows

(3) $${\text{PM}}_{2.5,\text{percentchange}}=(({\text{PM}}_{2.5,\text{endyear}}-{\text{PM}}_{2.5,\text{startyear}})/{\text{PM}}_{2.5,\text{startyear}})\times 100\%,$$

where PM_2.5_,_percentchange_ denoted the percent changes of PM_2.5_ during a study period. PM_2.5,endyear_ was the PM_2.5_ concentrations in the end year of the study period, and PM_2.5,startyear_ was the PM_2.5_ concentrations in the start year of the study period. Moreover, time-series analyses were conducted by year and season, respectively to quantitatively investigate the 10-year temporal trends of fine particle levels in the study domain and the Atlanta metro area.

## 3 Results

### 3.1 Descriptive statistics

The descriptive statistics of variables used in fitting the models are listed in Table 1. The annual mean PM_2.5_ concentrations ranged from 11.03 to 15.63 μg m^−3^ between 2001 and 2010, the highest occurring in 2005 and the lowest in 2009. The annual mean AOD values ranged from 0.20 to 0.28 during the same period of time. Table 1 also shows that land use variables and meteorological fields vary from year to year within the data.

### 3.2 Results of model-fitting and validation

**Figure 2:**
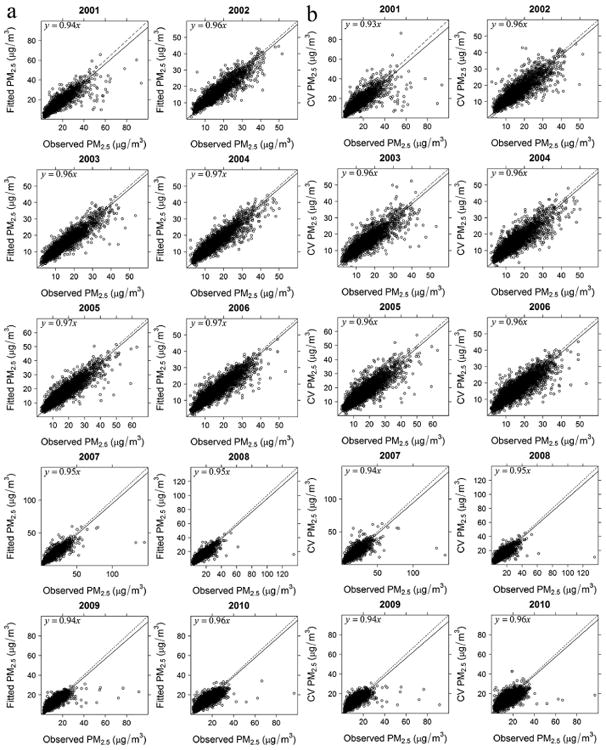
Model validation. **(a)** model fitting; **(b)** cross-validation.

The model-fitting and CV statistics (e.g., *R*^2^, MPE, and RM-SPE) are listed in Table 2. The results show that *R*^2^ ranges from 0.71 to 0.85, MPE is from 1.73 to 2.50 μgm^−3^, RM-SPE ranges from 2.75 to 4.10 μg m^−3^, and relative accuracy ranges from 72.9 to 80.7 %, which indicates a good fit between the predicted values from the fitted models and the observations. In addition, CV statistics results suggest that some model over-fitting is present; that is, *R*^2^ decreases, while MPE and RMSPE increase from model fitting to cross-validation, yet the differences are relatively small for all the years. For instance, *R*^2^ and relative accuracy have an average decrease of 0.08 and 4.21 %, respectively, while MPE and RMSPE have an average increase of 0.39 and 0.60 μgm^−3^, respectively, through all the years. Moreover, a regression of predicted values against the observations with an intercept at zero (Fig. 2) shows that, at high concentration levels, both model fitting and cross-validation under-predicted the PM_2.5_ concentrations by 3–7% (e.g., fitted/CV PM_2.5_ =97% to 93% observed PM_2.5_).

### 3.3 Spatial trends of PM_2.5_ concentrations

**Figure 3:**
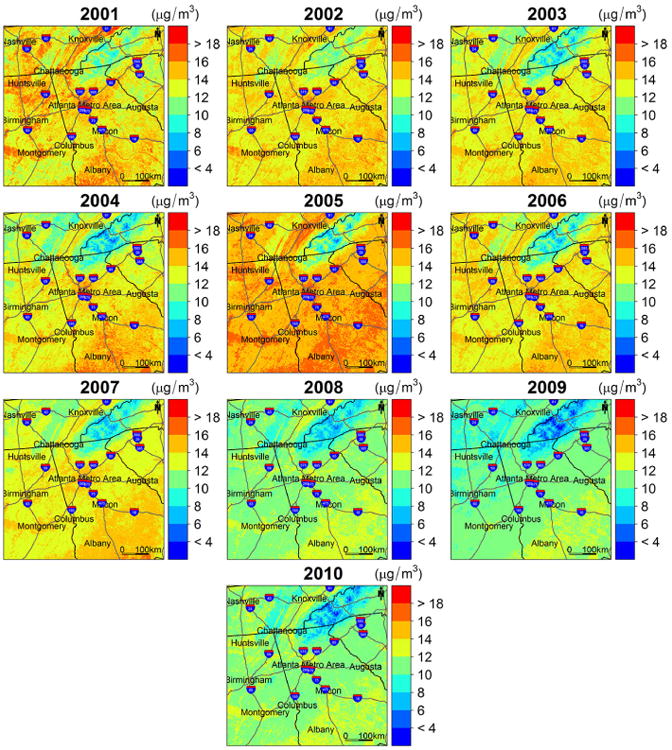
Annual mean PM_2.5_ concentration predictions in the study area.

**Figure 4:**
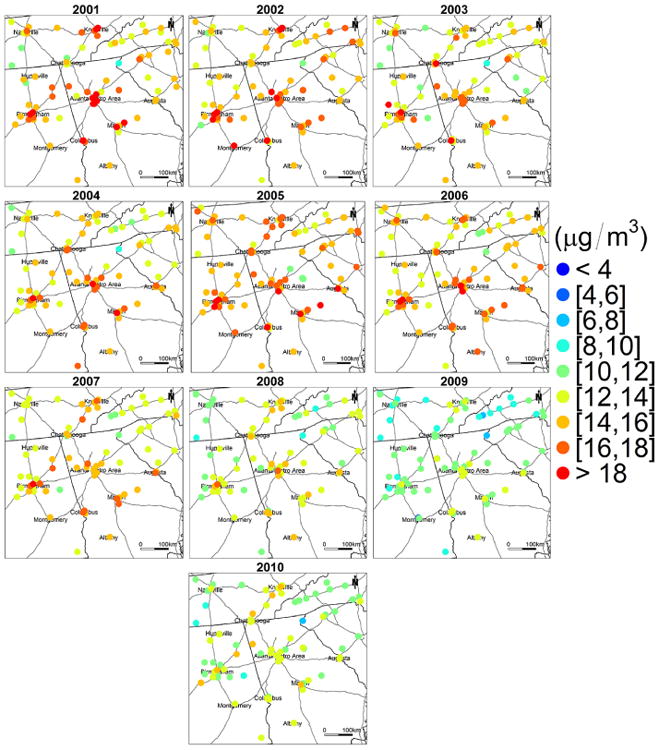
Annual mean PM_2.5_ concentration measured from ground FRM monitors.

**Figure 5:**
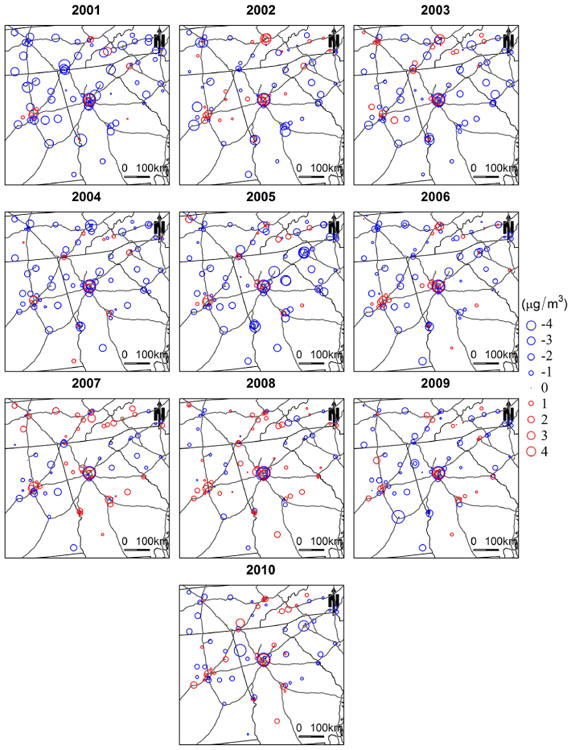
The differences between PM_2.5_ estimates and ground measurements at FRM monitors.

Figure 3 illustrates the PM_2.5_ concentration estimates at 1 km spatial resolution in the study area. The annual mean estimated concentrations are 13.97, 13.90, 13.35, 13.31, 15.19, 13.73, 13.22, 11.34, 10.58, and 11.22 μg m^−3^ for year 2001 though 2010, respectively. The spatial patterns of PM_2.5_ are very similar for all the years. High concentrations appear in large urban centers and along major highways, while low concentrations occur in rural and mountainous areas. In addition, high PM_2.5_ levels are also seen in the southeastern part of the study domain. Hu et al. (2014) reported elevated PM_2.5_ concentrations measured from monitoring sites located in this region. This area is primarily occupied by agriculture land, and high agricultural emissions may lead to elevated PM_2.5_ levels. As reported by previous studies, ammonia (NH_3_) and nitrogen oxides (NO_x_) generated by agricultural activities, such as farm vehicles, domestic and farm animals, and fertilizer applications, can significantly increase the number of suspended particles (Kurvits and Marta, 1998). However, specific agricultural emissions data are needed for further validation. In addition, biomass burning also contributes to emissions of fine particles in the region, following typical seasonal variations (Zhang et al., 2010). Figure 4 shows that the pattern of ground PM_2.5_ measurements from FRM monitors corresponds well with that of our estimated concentrations, and the differences between observed and estimated PM2.5 were within **±**3 μg m^−3^ for, on average, 92 % of the monitoring sites for the 10 years, indicating a good agreement between them (Fig. 5).

**Figure 6:**
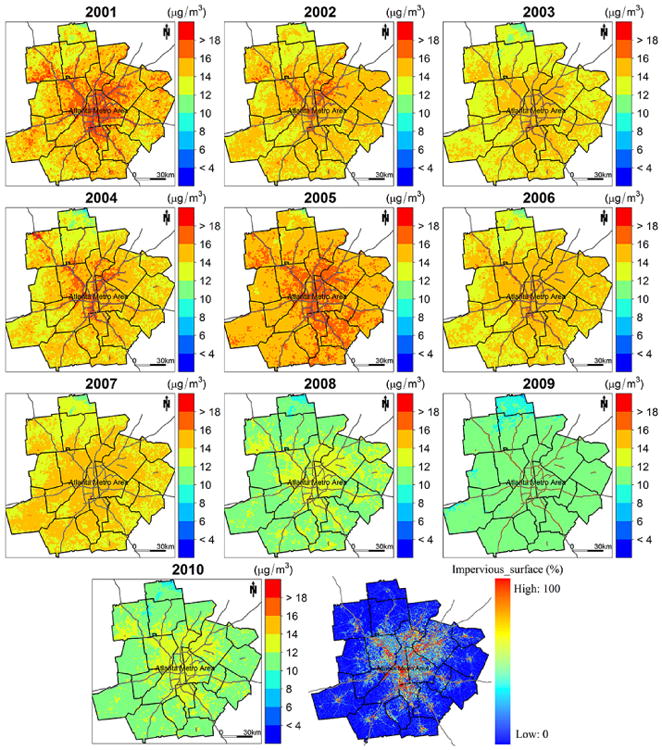
Annual mean PM_2.5_ concentration predictions in the Atlanta metro area.

To take advantage of the high spatial resolution of the MAIAC data, we generated a map of PM_2.5_ estimates in the Atlanta metro area for each year (Fig. 6). The annual mean estimates from 2001 to 2010 are 15.10, 14.64, 14.00, 14.54, 15.63, 14.39, 14.14, 11.78, 10.98, and 11.65 μgm^−3^, respectively. Compared to the last plot of Fig. 6, which illustrates the percentage of impervious surfaces and indicates the level of urban development, the PM_2.5_ maps distinctly show that high PM_2.5_ levels occur in areas with high urban land use and along major highways, while low concentrations appear in forest and recreational areas, suggesting an underlying positive relationship between air pollution levels and urban development.

**Figure 7:**
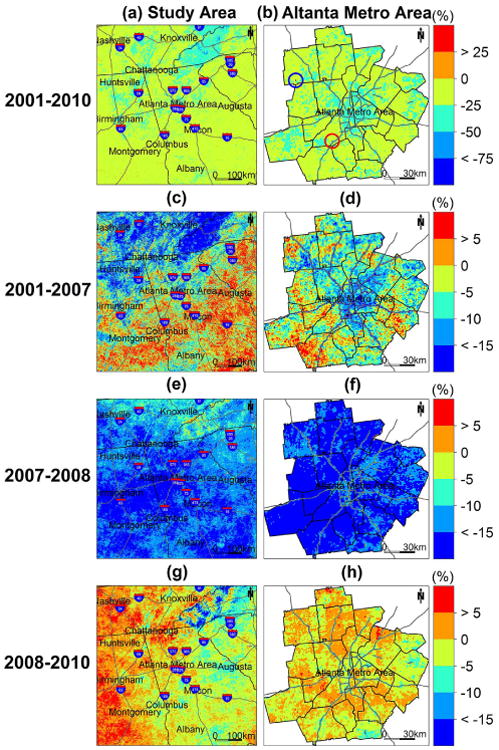
The percent changes of PM_2.5_ concentrations in the study area **(a)** and the Atlanta metro area **(b)** between 2001 and 2010, in the study area **(c)** and the Atlanta metro area **(d)** between 2001 and 2007, in the study area **(e)** and the Atlanta metro area **(f)** between 2007 and 2008, and in the study area **(g)** and the Atlanta metro area **(h)** between 2008 and 2010.

As shown in Fig. 7, PM_2.5_ concentrations have decreased on average ∼ 20 % for the entire domain and ∼23 % for the Atlanta metro area between 2001 and 2010. Figure 7a illustrates the spatial trend of changes in PM_2.5_ levels in the study region. The results show that PM_2.5_ levels in most of the areas decreased from 0 to 25 %, and large parts of the areas had decreases exceeding 25 % and as high as 50 %. Larger decreases occurred in more polluted areas such as the Atlanta metro area and along major highways, which might be due to recently enacted emission reduction programs (EPA, 2011) such as the EPA's Clean Air Interstate Rule (CAIR) issued in 2005 (http://www.epa.gov/cair/index.html), since the majority of emissions sources are located in or near urban areas and along major highways. Mitigation of fine particles has been effected by controlling direct PM_2.5_ emissions from both stationary and mobile sources (e.g., through installation of scrubbers and filters and the use of alternative fuels and electric vehicles) (EPA, 2007). The mountainous area in the northeastern part of our domain with generally low PM_2.5_ levels has also seen substantial decreases of PM_2.5_ concentrations. PM_2.5_ levels decreased from 25 to 50 % in most of the region, and some areas had decreases exceeding 50 %. By checking 2002 and 2008 point emissions data from the EPA NEI facility emissions reports (due to the lack of 2001 and 2010 data), the decreases are probably due to the dramatically reduced number of emission sources as well as the total emissions in the region during the period. Figure 7b illustrates the percent changes in PM_2.5_ levels within the Atlanta metro area. Once again, the spatial trend shows that larger decreases (25 to 50 %) primarily occurred in urban built-up areas and along major highways, while smaller decreases (0 to 25 %) appeared in forest or recreational areas with generally lower pollution levels. This result is expected because the changes of emissions mostly took place in urban built-up areas and along major highways. Two pixels with unusually large changes were identified (in blue and red circles). The large decrease (> 50 %) of PM_2.5_ concentration in the blue pixel was due to large emissions reduction from power plants located within that pixel during the period between 2001 and 2010. Likewise, the large increase (> 25 %) of PM_2.5_ concentration in the red pixel was due to the addition of a new emission source that did not exist in 2001.

We also illustrated the percent changes between 2001 and 2007, between 2007 and 2008, and between 2008 and 2010 in Fig. 7c–h, since the decreasing trend between 2001 and 2010 was nonlinear with small decreases between 2001 and 2007 (on average ∼5% for the entire domain and ∼6% for the Atlanta metro area) and between 2008 and 2010 (∼1% for both the entire domain and the Atlanta metro area) and a sharp decrease between 2007 and 2008 (∼14 % for the entire domain and ∼ 17 % for the Atlanta metro area). Figure 7c and d show that large decreases (> 10 %) between 2001 and 2007 mainly occurred in the northern part of the domain and the mountainous region as well as in urban built-up areas and along major highways in the Atlanta metro area, while increases (> 5 %) appeared in the southern and southeastern parts of our domain as well as in some residential and suburban regions in the Atlanta metro area. By comparing the 2002 with 2008 NEI point emissions data, this might be due to the addition of extra emissions sources in the region, despite the fact that total emissions dropped significantly during this period. Figure 7e and f show that large decreases (>10%) occurred in most of our domain between 2007 and 2008. We could not confirm whether this was related to emissions reductions in the absence of 2007 emissions data. Figure 7g and h illustrate the percent changes between 2008 and 2010. It revealed < 5 % increases in many areas in the eastern part of the domain with increases in some areas exceeding 5 %. On the other hand, many areas in the western part of the domain had < 5 % decreases with decreases in some parts of the mountainous region exceeding 15 %, In the Atlanta metro area, our results show decreases (< 10 %) in urban built-up areas and along major highways with some residential and suburban areas showing < 5% increases. Similarly, in the absence of 2010 emissions data, we could not examine whether these changes were associated with changes in emissions.

### 3.4 Temporal trends of PM_2.5_ concentrations

**Figure 8:**
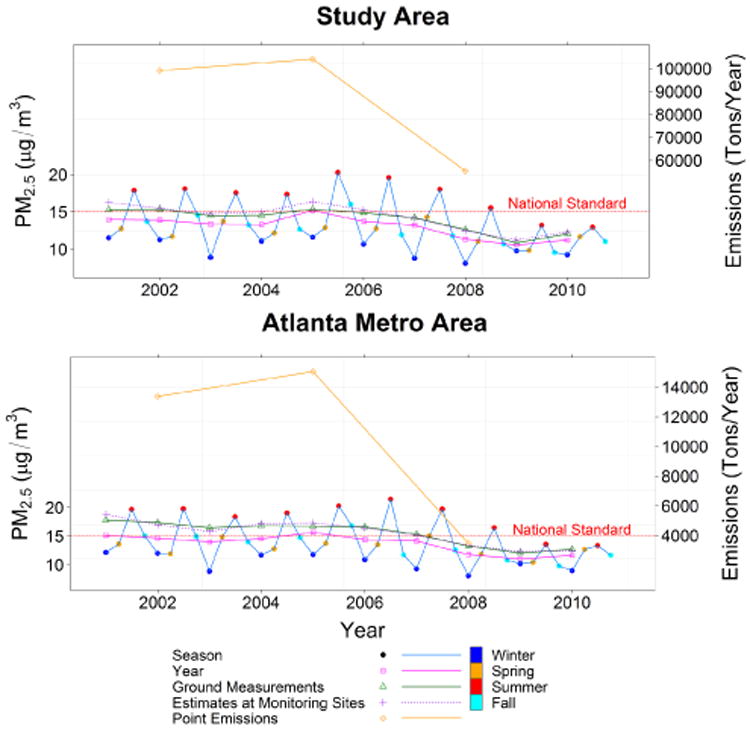
Time-series analyses of annual and seasonal mean PM_2.5_ concentrations and the point emissions from 2001 to 2010 for the study area and the Atlanta metro area.

A time-series analysis was conducted to quantitatively examine temporal trends of PM_2.5_ levels in the study area as well as the Atlanta metro area during the period between 2001 and 2010 (Fig. 8). The results show our model underestimated PM_2.5_ concentrations by 0.99 μg m^−3^ for the study domain and 1.82 μg m^−3^ for the Atlanta metro area. This is because satellite-estimated PM_2.5_ concentrations included both urban and rural regions, while the ground measurements mostly represented urban conditions. On the other hand, our estimates over monitoring sites matched well with the ground measurements. The mean difference was 0.4μg m^−3^ for the Atlanta metro area and 0.41 μg m^−3^ for the study domain. The PM_2.5_ levels in the study region as well as the Atlanta metro area had relatively small fluctuations from 2001 to 2007 and from 2008 to 2010, while there was a significant drop in 2008, which was probably due to significant emissions reduction in 2008. The results also reveal seasonal variations of PM_2.5_ levels with the highest concentrations occurring in summer and the lowest appearing in winter. Between 2001 and 2010, our time-series analysis showed that the annual mean PM_2.5_ concentration decreased ∼ 20 % in the study area and ∼23 % in the Atlanta metro area, which is in line with the findings documented in the US EPA report on particle pollution (EPA, 2011). Both the EPA's findings and our results illustrate a peak in PM_2.5_ levels in year 2005, and this phenomenon might be attributed to the increase of sulfate concentrations emitted from electric utilities and industrial boilers during the warm months (e.g, from May to September) of 2005 (EPA, 2008b). In addition, the decrease of PM_2.5_ levels after year 2005 likely is due to the emissions reduction programs that have been enacted recently (EPA, 2007, EPA, 2011) such as the EPA's CAIR issued in 2005. Figure 8 distinctly shows this sharp decrease of emissions from 2005 to 2008. In addition, the sharp decrease from 2007 to 2008 also may be partially attributed to the national financial crisis starting in late 2007. The economic slowdown had clear impacts on manufacturing productivity (e.g., the real gross domestic product (GDP) of metro Atlanta in the manufacturing sector dropped 10.2% from 2007 to 2008 (www.bea.gov)), and may also have led to decreases in PM_2.5_ emissions.

## 4 Discussion

A major strength of this study is that we used high-resolution PM_2.5_ estimates derived from MAIAC AOD to investigate spatiotemporal trends of PM_2.5_ concentrations in the study area. PM_2.5_ estimates at finer resolutions are more suitable for investigation of spatial trends than those at coarser resolutions derived from other AOD products (e.g., MODIS and MISR), because estimates at coarser scales inevitably omit local spatial details, as pointed out by Hu et al. (2014). Our results are capable of showing PM_2.5_ concentrations and changes at 1 km resolution, which are very useful for air pollution studies at local scales. For instance, spatial trends of changes in PM_2.5_ concentrations in the Atlanta metro area show greater PM_2.5_ reduction in more polluted areas (e.g., urban built-up areas and along major highways). Some of the changes may be directly associated with the addition or removal of one or more emission sources as well as the increase or decrease of emissions from those sources. Although high-resolution PM_2.5_ estimates can provide more details to examine spatial trends, difficulties lie in their validation to ground monitoring. More ground measurements at specific locations are needed to further validate the results.

Our results of temporal trends of PM_2.5_ concentrations correspond well with the EPA's results (EPA, 2011). However, our results show that both ground measurements and satellite-estimated PM_2.5_ concentrations over the monitoring sites were generally higher than PM_2.5_ estimates over the entire study domain. This is because most of the EPA FRM monitors are located in or near urban areas with generally higher PM_2.5_ levels, and therefore observed and satellite-estimated PM_2.5_ levels over the monitoring sites reflect mostly urban conditions. Conversely, PM_2.5_ estimates over the entire study area account for both urban and rural areas, and therefore the temporal trends of satellite-estimated PM_2.5_ concentrations over the entire study domain might be more representative of the true fluctuations of regional PM_2.5_ levels. Our future research will continue to explore these associations.

A limitation of our study was that only 3 years of emissions data (2002, 2005, and 2008) were available. The NEI is prepared every 3 years by the EPA primarily based on emissions estimates, emissions model inputs, and supplementary data. As a result, a more quantitative comparison between estimated PM_2.5_ and emissions could not be conducted.

We realize that both meteorological fields and land use variables can be potentially incorporated in the second stage GWR model. The primary objective of this study was to investigate the spatial and temporal trends of PM_2.5_ levels in the southeastern US. Hence, it was not pursued in this study and will be addressed in future research.

## 5 Conclusions

In this paper, we used a two-stage spatiotemporal model incorporating MAIAC AOD data, meteorological fields, and land use variables to estimate PM_2.5_ concentrations at 1 km spatial resolution and investigated the 10-year spatial and temporal trends of PM_2.5_ levels in the southeastern US. As expected, the satellite model predicted high concentrations in large urban centers and along major highways and low concentrations in rural and mountainous area with relatively high accuracy. Our time-series analysis results indicate that the PM_2.5_ levels decreased ∼20 % in the study region and ∼23 % in the Atlanta metro area during the period between 2001 and 2010, and the largest drop occurred between 2007 and 2008. More polluted areas (e.g., in urban areas and along major highways) have also seen greater reduction in PM_2.5_ levels, while forests and recreational areas had lower and moderate reduction. PM_2.5_ estimates at high spatial resolutions can provide more details in small geographic regions and may reduce exposure misclassification in air pollution and epidemiological studies. High-resolution PM_2.5_ data are also useful for air pollution monitoring in large geographic areas because they can not only greatly expand the spatial coverage of costly ground monitoring networks, but they also provide more accurate estimates of population exposure to PM_2.5_. In addition, regional transport of PM_2.5_ can be better examined via comparing the spatial distributions of daily high-resolution PM_2.5_ estimates, raising new questions for future research.

## Supplementary Material

2

## Figures and Tables

**Table 1:** Descriptive statistics (2001–2010).

Var.	Min	SD	Max	Mean
PM_2.5_ (μgm^−3^**)**	2.0–2.6	5.31–8.64	50.1–145.0	11.03–15.63
Boundary layer height (m)	215–464	347–493	2605–3405	1146–1464
Relative humidity (%)	13.9–26.2	8.7–11.3	86.8–93.1	46.8–59.9
*U* wind (m s^−1^)	−9.44 to −6.30	2.62–3.20	10.22–16.85	0.82–1.47
*V* wind (ms^−1^)	−12.60 to −9.34	2.62–3.00	8.45–11.84	-0.74 to -0.09
Wind speed (ms^−1^)	0.03–0.12	1.81–2.13	12.76–18.06	3.48–3.99
Forest cover 2001	0	0.16–0.18	0.83	0.14–0.17
Forest cover 2006	0	0.15–0.17	0.79	0.14–0.16
Road length (m)	0	187.29–230.81	1012.97–1078.09	58.05–82.92
Elevation (m)	46.78	126.82–141.65	811.63–822.82	227.74–249.10
Point emissions 2002 (tons year^−1^)	0	56.64–70.39	364.42	11.13–16.46
Point emissions 2005 (tons year^−1^)	0	150.89–188.63	985.48	26.90–40.84
Point emissions 2008 (tons year^−1^)	0	15.89–19.72	101.74	3.14–4.54
AOD	0–0.01	0.16–0.26	1.42–1.96	0.20–0.28

**Table 2:** Model validation.

Year			Model fitting				Cross-validation	
	*R*^2^	MPE(μgm^−3^**)**	RMSPE(μgm^−3^**)**	Relative accuracy (%)*	*R*^2^	MPE(μgm^−3^)	RMSPE(μgm^−3^)	Relative accuracy (%)*
2001	0.78	2.50	4.10	72.9	0.67	3.01	5.00	67.0
2002	0.84	2.10	2.98	80.7	0.75	2.62	3.75	75.7
2003	0.85	1.95	2.77	80.4	0.76	2.42	3.47	75.4
2004	0.85	1.97	2.77	80.3	0.77	2.40	3.37	76.1
2005	0.84	2.23	3.17	79.7	0.78	2.64	3.76	75.9
2006	0.85	2.02	2.90	80.6	0.78	2.43	3.49	76.6
2007	0.79	2.26	3.75	74.0	0.71	2.64	4.39	69.6
2008	0.74	1.93	3.13	75.4	0.67	2.21	3.53	72.3
2009	0.71	1.73	2.88	73.9	0.62	2.00	3.28	70.3
2010	0.73	1.90	2.75	77.6	0.66	2.15	3.12	74.5

* Relative accuracy is defined as 100% – RMSPE/the mean PM_2.5_ concentration.

## References

[R1] Chudnovsky AA, Kostinski A, Lyapustin A, Koutrakis P (2012). Spatial scales of pollution from variable resolution satellite imaging. Environ Pollut.

[R2] Crouse DL, Peters PA, van Donkelaar A, Goldberg MS, Villeneuve PJ, Brion O, Khan S, Atari DO, Jerrett M, Pope CA, Brauer M, Brook JR, Martin RV, Stieb D, Burnett RT (2012). Risk of Non accidental and Cardiovascular Mortality in Relation to Long-term Exposure to Low Concentrations of Fine Particulate Matter: A Canadian National-Level Cohort Study. Environ Health Persp.

[R3] (2007). EPA: lists of potential control measures for PM_2.5_ and precursors, Draft Version 1.0. http://www.epa.gov/pm/measures/pm_control_measures_tables_ver1.pdf.

[R4] EPA (2008a). Quality assurance handbook for air pollution measurement systems, Vollume II, Ambient Air Quality Monitoring Program.

[R5] EPA (2008b). National Air Quality – Status and Trends through 2007. US Environmental Protection Agency.

[R6] EPA (2011). National Air Quality – Status and Trends through 2010. US Environmental Protection Agency.

[R7] Gupta P, Christopher SA (2009). Particulate matter air quality assessment using integrated surface, satellite, and meteorological products: Multiple regression approach. J Geophys Res-Atmos.

[R8] Hu X, Waller LA, Al-Hamdan MZ, Crosson WL, Estes MG, Estes SM, Quattrochi DA, Sarnat JA, Liu Y (2013). Estimating ground-level PM_2.5_ concentrations in the southeastern U.S. using geographically weighted regression. Environ Res.

[R9] Hu X, Waller LA, Lyapustin A, Wang Y, Al-Hamdan MZ, Crosson WL, Estes MG, Estes SM, Quattrochi DA, Puttaswamy SJ, Liu Y (2014). Estimating ground-level PM_2.5_ concentrations in the Southeastern United States using MAIAC AOD retrievals and a two-stage model. Remote Sens Environ.

[R10] Ito K, Thurston GD, Silverman RA (2007). Characterization of PM_2.5_, gaseous pollutants, and meteorological interactions in the context of time-series health effects models. J Expo Sci Env Epid.

[R11] Kloog I, Koutrakis P, Coull BA, Lee HJ, Schwartz J (2011). Assessing temporally and spatially resolved PM_2.5_ exposures for epidemiological studies using satellite aerosol optical depth measurements. Atmos Environ.

[R12] Kurvits T, Marta T (1998). Agricultural NH_3_ and NO_x_ emissions in Canada. Environ Pollut.

[R13] Lee HJ, Liu Y, Coull BA, Schwartz J, Koutrakis P (2011). A novel calibration approach of MODIS AOD data to predict PM_2.5_ concentrations. Atmos Chem Phys.

[R14] Lee HJ, Coull BA, Bell ML, Koutrakis P (2012). Use of satellite-based aerosol optical depth and spatial clustering to predict ambient PM_2.5_ concentrations. Environ Res.

[R15] Liu Y, Franklin M, Kahn R, Koutrakis P (2007). Using aerosol optical thickness to predict ground-level PM_2.5_ concentrations in the St. Louis area: A comparison between MISR and MODIS. Remote Sens Environ.

[R16] Liu Y, Paciorek CJ, Koutrakis P (2009). Estimating Regional Spatial and Temporal Variability of PM_2.5_ Concentrations Using Satellite Data, Meteorology, and Land Use Information. Environ Health Persp.

[R17] Lyapustin A, Martonchik J, Wang YJ, Laszlo I, Korkin S (2011a). Multiangle implementation of atmospheric correction (MAIAC): 1. Radiative transfer basis and look-up tables. J Geophys Res-Atmos.

[R18] Lyapustin A, Wang Y, Laszlo I, Kahn R, Korkin S, Remer L, Levy R, Reid JS (2011b). Multiangle implementation of atmospheric correction (MAIAC): 2. Aerosol algorithm. J Geophys Res-Atmos.

[R19] Lyapustin A, Wang Y, Laszlo I, Hilker T, Hall FG, Sellers PJ, Tucker CJ, Korkin SV (2012). Multi-angle implementation of atmospheric correction for MODIS (MAIAC): 3 Atmospheric correction. Remote Sens Environ.

[R20] Mesinger F, DiMego G, Kalnay E, Mitchell K, Shafran PC, Ebisuzaki W, Jovic D, Woollen J, Rogers E, Berbery EH (2006). North American regional reanalysis. B Am Meteorol Soc.

[R21] Paciorek CJ, Liu Y, Moreno-Macias H, Kondragunta S (2008). Spatiotemporal associations between GOES aerosol optical depth retrievals and ground-level PM_2.5_. Environ Sci Technol.

[R22] Peng RD, Bell ML, Geyh AS, McDermott A, Zeger SL, Samet JM, Dominici F (2009). Emergency Admissions for Cardiovascular and Respiratory Diseases and the Chemical Composition of Fine Particle Air Pollution. Environ Health Persp.

[R23] Prados AI, Kondragunta S, Ciren P, Knapp KR (2007). GOES Aerosol/Smoke Product (GASP) over North America: Comparisons to AERONET and MODIS observations. J Geophys Res.

[R24] Schafer K, Harbusch A, Emeis S, Koepke P, Wiegner M (2008). Correlation of aerosol mass near the ground with aerosol optical depth during two seasons in Munich. Atmos Environ.

[R25] So KL, Guo H, Li YS (2007). Long-term variation of PM_2.5_ levels and composition at rural, urban, and roadside sites in Hong Kong: Increasing impact of regional air pollution. Atmos Environ.

[R26] van Donkelaar A, Martin RV, Brauer M, Kahn R, Levy R, Verduzco C, Villeneuve PJ (2010). Global Estimates of Ambient Fine Particulate Matter Concentrations from Satellite-Based Aerosol Optical Depth: Development and Application. Environ Health Persp.

[R27] Wallace J, Kanaroglou P, Ieee (2007). An investigation of air pollution in southern Ontario, Canada, with MODIS and MISR aerosol data, in: Igarss: 2007 Ieee International Geoscience and Remote Sensing Symposium, Vols 1–12 – Sensing and Understanding Our Planet. IEEE International Symposium on Geo-science and Remote Sensing (IGARSS).

[R28] Weber R, Orsini D, Duan Y, Baumann K, Kiang CS, Chameides W, Lee YN, Brechtel F, Klotz P, Jongejan P, ten Brink H, Slanina J, Boring CB, Genfa Z, Dasgupta P, Hering S, Stolzenburg M, Dutcher DD, Edgerton E, Hartsell B, Solomon P, Tanner R (2003). Intercomparison of near real time monitors of PM_2.5_ nitrate and sulfate at the US Environmental Protection Agency Atlanta Supersite. J Geophys Res-Atmos.

[R29] Zeger SL, Thomas D, Dominici F, Samet JM, Schwartz J, Dockery D, Cohen A (2000). Exposure measurement error in time-series studies of air pollution: concepts and consequences. Environ Health Persp.

[R30] Zhang X, Hecobian A, Zheng M, Frank NH, Weber RJ (2010). Biomass burning impact on PM_2.5_ over the southeastern US during 2007: integrating chemically speciated FRM filter measurements, MODIS fire counts and PMF analysis. Atmos Chem Phys.

[R31] Zhang Y, Yu H, Eck TF, Smirnov A, Chin M, Remer LA, Bian H, Tan Q, Levy R, Holben BN, Piazzolla S (2012). Aerosol daytime variations over North and South America derived from multiyear AERONET measurements. J Geophys Res-Atmos.

