# Supplementary material for: 10-year spatial and temporal trends of PM2.5 concentrations in the southeastern US estimated using high-resolution satellite data
Source: Atmos Chem Phys. Author manuscript; Available in PMC 2017 Sep 28. (PMC5619667; doi:10.5194/acp-14-6301-2014)
Supplement: 2 [file NIHMS899888-supplement-2.pdf]

Supplement of Atmos. Chem. Phys., 14, 6301–6314, 2014
http://www.atmos-chem-phys.net/14/6301/2014/
doi:10.5194/acp-14-6301-2014-supplement
© Author(s) 2014. CC Attribution 3.0 License.

Atmospheric Chemistry and Physics
Open Access

Supplement of

10-year spatial and temporal trends of $PM_{2.5}$ concentrations in the southeastern US estimated using high-resolution satellite data

X. Hu et al.

Correspondence to: Y. Liu (yang.liu@emory.edu)

Table S1. Model structures.

Year	Model
2001	$PM_{2.5,st} = (\beta_0 + \beta_{0,t}) + (\beta_1 + \beta_{1,t})AOD_{st} + (\beta_2 + \beta_{2,t})Boundary\ Layer\ Height_{st} + (\beta_3 + \beta_{3,t})Wind\ Speed_{st} +$ $\beta_4 Elevation_s + \beta_5 MajorRoads_s + \beta_6 Forest\ Cover2001_s + \beta_7 Point\ Emissions2002_s + \varepsilon_{st}$ $(\beta_{0,t}\beta_{1,t}\beta_{2,t}\beta_{3,t}) \sim N[(0,0,0,0),\Psi]$
2002	$PM_{2.5,st} = (\beta_0 + \beta_{0,t}) + (\beta_1 + \beta_{1,t})AOD_{st} + (\beta_2 + \beta_{2,t})RelativeHumidity_{st} + (\beta_3 + \beta_{3,t})U - Wind_{st} +$ $(\beta_4 + \beta_{4,t})V - Wind_{st} + \beta_5 Elevation_s + \beta_6 MajorRoad_s + \beta_7 Forest\ Cover2001_s +$ $\beta_8 Point\ Emissions2002_s + \varepsilon_{st}$ $(\beta_{0,t}\beta_{1,t}\beta_{2,t}\beta_{3,t}\beta_{4,t}) \sim N[(0,0,0,0,0),\Psi]$
2003	$PM_{2.5,st} = (\beta_0 + \beta_{0,t}) + (\beta_1 + \beta_{1,t})AOD_{st} + (\beta_2 + \beta_{2,t})Relative\ Humidity_{st} + (\beta_3 + \beta_{3,t})U - Wind_{st} +$ $(\beta_4 + \beta_{4,t})V - Wind_{st} + \beta_5 Elevation_s + \beta_6 Major\ Roads_s + \beta_7 Forest\ Cover2001_s +$ $\beta_8 Point\ Emissions2002_s + \varepsilon_{st}$ $(\beta_{0,t}\beta_{1,t}\beta_{2,t}\beta_{3,t}\beta_{4,t}) \sim N[(0,0,0,0,0),\Psi]$
2004	$PM_{2.5,st} = (\beta_0 + \beta_{0,t}) + (\beta_1 + \beta_{1,t})AOD_{st} + (\beta_2 + \beta_{2,t})Relative\ Humidity_{st} + (\beta_3 + \beta_{3,t})U - Wind_{st} +$ $(\beta_4 + \beta_{4,t})V - Wind_{st} + \beta_5 Elevation_s + \beta_6 Major\ Roads_s + \beta_7 Forest\ Cover2006_s +$ $\beta_8 Point\ Emissions2005_s + \varepsilon_{st}$ $(\beta_{0,t}\beta_{1,t}\beta_{2,t}\beta_{3,t}\beta_{4,t}) \sim N[(0,0,0,0,0),\Psi]$
2005	$PM_{2.5,st} = (\beta_0 + \beta_{0,t}) + (\beta_1 + \beta_{1,t})AOD_{st} + (\beta_2 + \beta_{2,t})Relative\ Humidity_{st} + (\beta_3 + \beta_{3,t})Wind\ Speed_{st} +$ $\beta_4 Elevation_s + \beta_5 Major\ Roads_s + \beta_6 Forest\ Cover2006_s + \beta_7 Point\ Emissions2005_s + \varepsilon_{st}$ $(\beta_{0,t}\beta_{1,t}\beta_{2,t}\beta_{3,t}) \sim N[(0,0,0,0),\Psi]$
2006	$PM_{2.5,st} = (\beta_0 + \beta_{0,t}) + (\beta_1 + \beta_{1,t})AOD_{st} + (\beta_2 + \beta_{2,t})Relative\ Humidity_{st} + (\beta_3 + \beta_{3,t})U - Wind_{st} +$ $(\beta_4 + \beta_{4,t})V - Wind_{st} + \beta_5 Elevation_s + \beta_6 Major\ Roads_s + \beta_7 Forest\ Cover2006_s +$ $\beta_8 Point\ Emissions2005_s + \varepsilon_{st}$ $(\beta_{0,t}\beta_{1,t}\beta_{2,t}\beta_{3,t}\beta_{4,t}) \sim N[(0,0,0,0,0),\Psi]$
2007	$PM_{2.5,st} = (\beta_0 + \beta_{0,t}) + (\beta_1 + \beta_{1,t})AOD_{st} + (\beta_2 + \beta_{2,t})Relative\ Humidity_{st} + (\beta_3 + \beta_{3,t})Wind\ Speed_{st} +$ $\beta_4 Elevation_s + \beta_5 MajorRoads_s + \beta_6 Forest\ Cover2006_s + \beta_7 Point\ Emissions2008_s + \varepsilon_{st}$ $(\beta_{0,t}\beta_{1,t}\beta_{2,t}\beta_{3,t}) \sim N[(0,0,0,0),\Psi]$
2008	$PM_{2.5,st} = (\beta_0 + \beta_{0,t}) + (\beta_1 + \beta_{1,t})AOD_{st} + (\beta_2 + \beta_{2,t})Relative\ Humidity_{st} + (\beta_3 + \beta_{3,t})Wind\ Speed_{st} +$ $\beta_4 Elevation_s + \beta_5 Major\ Roads_s + \beta_6 Forest\ Cover2006_s + \beta_7 Point\ Emissions2008_s + \varepsilon_{st}$ $(\beta_{0,t}\beta_{1,t}\beta_{2,t}\beta_{3,t}) \sim N[(0,0,0,0),\Psi]$

2009	$PM_{2.5,st} = (\beta_0 + \beta_{0,t}) + (\beta_1 + \beta_{1,t}) AOD_{st} + (\beta_2 + \beta_{2,t}) Relative\ Humidity_{st} + (\beta_3 + \beta_{3,t}) Wind\ Speed_{st} +$ $\beta_4 Elevation_s + \beta_5 Major\ Roads_s + \beta_6 Forest\ Cover2006_s + \beta_7 Point\ Emissions2008_s + \varepsilon_{st}$ $(\beta_{0,t} \beta_{1,t} \beta_{2,t} \beta_{3,t}) \sim N[(0,0,0,0),\Psi]$
2010	$PM_{2.5,st} = (\beta_0 + \beta_{0,t}) + (\beta_1 + \beta_{1,t}) AOD_{st} + (\beta_2 + \beta_{2,t}) Boundary\ Layer\ Height_{st} + (\beta_3 + \beta_{3,t}) U - Wind_{st} +$ $(\beta_4 + \beta_{4,t}) V - Wind_{st} + \beta_5 Elevation_s + \beta_6 Forest\ Cover2006_s +$ $\beta_7 Point\ Emissions2008_s + \varepsilon_{st}$ $(\beta_{0,t} \beta_{1,t} \beta_{2,t} \beta_{3,t} \beta_{4,t}) \sim N[(0,0,0,0,0),\Psi]$
